# AI biases as asymmetries: a review to guide practice

**DOI:** 10.3389/fdata.2025.1532397

**Published:** 2025-10-13

**Authors:** Gabriella Waters, Phillip Honenberger

**Affiliations:** Center for Equitable AI and Machine Learning Systems (CEAMLS), Morgan State University, Baltimore, MD, United States

**Keywords:** bias, artificial intelligence, machine learning, symmetry, statistical bias, cognitive bias, inductive bias, bias-variance trade-off

## Abstract

The understanding of bias in AI is currently undergoing a revolution. Often assumed to be errors or flaws, biases are increasingly recognized as integral to AI systems and sometimes preferable to less biased alternatives. In this paper we review the reasons for this changed understanding and provide new guidance on three questions: First, how should we think about and measure biases in AI systems, consistent with the new understanding? Second, what kinds of bias in an AI system should we accept or even amplify, and why? And, third, what kinds should we attempt to minimize or eliminate, and why? In answer to the first question, we argue that biases are “violations of a symmetry standard” (following Kelly). Per this definition, many biases in AI systems are benign. This raises the question of how to identify biases that *are* problematic or undesirable when they occur. To address this question, we distinguish three main ways that asymmetries in AI systems can be problematic or undesirable—erroneous representation, unfair treatment, and violation of process ideals—and highlight places in the pipeline of AI development and application where bias of these types can occur.

## 1 Introduction

The understanding of bias in AI is currently undergoing a revolution. Often perceived as errors or flaws, biases are increasingly recognized as integral to AI systems and sometimes preferable to less biased alternatives (Mitchell, 1980; Geman et al., 1992; Yarkoni and Westfall, 2017; Danks and London, 2017; Taniguchi et al., 2018; Montanez et al., 2019). Cognitive psychology and statistics have informed this shift, highlighting not only the costs, but also the circumstantial benefits of biases in decision-making processes. Cognitive psychology, for instance, presents biases as often helpful in making decisions under conditions of uncertainty (Tversky and Kahneman, 1974; Gigerenzer and Brighton, 2009; Hafenbraedl et al., 2016; Taniguchi et al., 2018). Similarly, statistical methods acknowledge biases as often useful and sometimes necessary for making inferences from data (Mitchell, 1980; Geman et al., 1992; Yarkoni and Westfall, 2017; Montanez et al., 2019). These insights have been instrumental in redefining biases as not inherently negative, but as sometimes essential components that can and should be harnessed to improve AI systems.

This new perspective on AI biases raises several important questions, including: (1) How should we think about and measure biases in AI systems, consistent with the new understanding? (2) What kinds of bias in an AI system should we accept or even amplify, and why? and (3) which biases should we criticize, avoid, or eliminate, and why?

This paper makes four major contributions on this topic (summarized in Table 1). First, we review prior efforts to define AI biases, highlighting their weaknesses, and present a thorough articulation and defense of a new comprehensive definition of bias in AI systems: the “bias as asymmetry” definition (Section 3). Second, we provide further evidence and argument for the emerging view that biases in AI systems are not all bad (Sections 2 and 7). Thirdly and perhaps most importantly, we provide a principled and widely applicable answer to an important question raised by the “AI biases are not all bad” perspective: “*When* are AI biases bad or not bad, and why?” (Sections 4–8 and Table 2). Fourth and finally, we rely on our answers to this question to make recommendations for response to AI biases of various types (Sections 8–9, Table 2).

**Table 1:** Contents of article, organized by four primary contributions and two incidental contributions.

**Section of paper**	**Primary contributions**				**Incidental contributions**	
	**Defining “bias”**	**Establishing that not all AI biases are unacceptable**	**Clarifying which AI biases are acceptable and which are unacceptable, and why**	**Recommending responses to AI biases of various types**	**Reinterpreting well-known cases of AI bias**	**Reinterpreting bias in LLMs**
Section 1						
Section 2						
Section 3						
Section 4						
Section 5						
Section 6						
Section 7						
Section 8						
Section 9						
Section 10						
Section 11						
Section 12						
Table 2						

Shading indicates which parts of the paper (vertical axis) are devoted to which contributions (horizontal axis).

**Table 2:** An overview of major *AI biases* and associated *examples, type of asymmetry exhibited, and recommended mitigations* (horizontal axis), ordered by the *stage of AI development cycle in which they appear* (vertical axis).

**Lifecycle component**	**Agent**	**Relevant bias types (partial list)**	**Example**	**Axis of asymmetry**	**Possible mitigations/recommended responses**
(a) How model is chosen	Human	Inductive bias	A human selects a particular k for application of a k-means unsupervised classifier	Between one inductive procedure or model architecture consistent with the data, and all possible inductive procedures or model architectures consistent with the data	Possibly acceptable bias. Researchers should reflect on the effects of any inductive biases and experiment with and explore the results from different sets of inductive assumptions
		Availability heuristic	A human gives an image recognition problem to a LLM they often use for translation tasks, even though a supervised classifier would be more accurate	Favored selection of *familiar* tools or procedures, among possible tools or procedures	Generally unacceptable bias, except when (a) tradeoffs between the accuracy costs of the bias and benefits to other features of importance to the decision-making situation (e.g. speed, energetic cost) justify the lower accuracy, or (b) the *particular* familiar tool happens to be among the most accurate for the task. In the second case, the bias is still a process bias that is concerning, given its likely overall cost in the long run of scenario-diverse applications
(b) How data are collected	Human or AI or human-AI hybrid	Historical bias	A loan approval algorithm is trained on U.S. loan repayment data from 1931 to 2023, without consideration of how repayment may be affected by a financial regime discriminatory against non-white and non-male borrowers	(a) Between groups within historical data; and (b) between reliance on historical data and reliance on data better indicative of possible or future reality	Identify the purpose for which the data will be used. If the purpose is to accurately represent the past, the bias is acceptable. If the purpose is to predict the future, the bias may be unfair and unacceptable; possible mitigations include *post-hoc* corrections to bring dataset into better alignment with current and projected near-future conditions and/or fairness constraints
		Label bias	The sentiment labels chosen for images are influenced by an annotator's mood or culture	Process bias. Labeling practice deviates in a regular way (due to factors such as emotion or cultural background) from a labeling ideal of non-partiality and objectivity (b) Inequality bias. Axis of asymmetry is between labels in different parts of the dataset, due to different labelers and their backgrounds	Reduce extent of bias by providing operational criteria for labeling practices to labelers. Document, analyze, and report on statistical features of labeling practices. Supply maximum metadata about data labeling practices (so that any biases in labeling are more likely to be identified and can be corrected for) *Post-hoc* relabeling. *Post-hoc* error correction
		Sampling bias	Over 50% of the images collected for training a facial recognition algorithm are of Caucasian faces, despite Caucasians making up only 10%−25% of the world population (cf. Buolamwini and Gebru, 2018)	(a) Between how much data is collected for certain groups or types of objects; and how much data is collected for other groups or types of objects; (b) between the *representativeness of the quantity of data* when compared across groups	Generally unacceptable. Restart from a more representative dataset through (a) finding a more balanced dataset already in existence, (b) acquiring more real data of the types needed to balance the current dataset (without generating new problematic biases in the data acquisition procedure!), (c) generating synthetic data of the types needed to balance the current dataset. Alternatively: Restrict conclusions of analysis (including decision support) to whatever populations the dataset is actually representative of (e.g. English-language text outputs for an overwhelmingly English-language-trained LLM)
		Feedback bias	An LLM is trained to answer geography questions partly via data produced by prior LLM outputs, thus magnifying the effects of hallucinations and Anglocentric biases in these prior outputs	(a1) Process bias because geography data is not drawn from human-produced sources (as assumed) but also from prior AI summaries and hence amplification of *some* aspects of the human-produced sources asymmetrically by comparison to other aspects. In addition: (a2) Error biases may be introduced by amplification of hallucinations. Asymmetry is between an ideally faithful report on human geography and the actual (erroneous) report from the model. (a3) Potentially unfair inequality bias produced by amplification of Anglophone bias. Asymmetry is between Anglophone labels and demarcations, on the one hand, and those recognized elsewhere, on the other	(a) Train with a more carefully curated dataset wherein systematic errors and biases in the dataset have been systematically removed
			(b) A recommender system that is informed by user behavior offers recommendations, which lead to narrowed user behavior, which leads to narrower recommendations from the recommender system, which leads to even more narrowed user behavior	(b1) Error bias because the ultimate set of recommendations is narrower than the user would probably prefer (assuming “actual user preference” as the ground truth)(b2) Inequality bias because prior user behavior shaped by the system is given a disproportionate influence, by comparison with other aspects of user behavior. (b3) The inequality bias in (b2) constitutes a process bias insofar as this process of selecting recommendations deviates from a more balanced and diverse ideal.	(b) In circumstances where misaligned feedback loops can occur, flag of possible feedback-affected pathways and make associated adjustments to weights at the in-processing stage.
(c) How much representative data is collected	Human or AI or human-AI hybrid	Sampling bias	(see above)	Between how much data is collected for certain groups or types of objects, and how much data is collected for other groups or types of objects.	(see above)
(d) How accurately the data represent	Data	Error bias	Ecological data were collected by four field workers, one of whom was unreliable and produced systematically inflated values for some parameters	(a) Process bias. Some data were collected in a manner that violates reliable data collection standards. (b) Error bias. Roughly $\frac{1}{4}$ of the data exhibits artificially inflated values, which changes the top-level statistical features of the dataset so that it becomes a less accurate measure of these features than it would otherwise be. The axis of asymmetry is between the dataset and its recorded values, on the one hand, and the ecological reality this dataset and its values supposedly represents, on the other	Generally unacceptable. Erroneous or unreliable data can be thrown out (so long as this omission doesn't introduce new biases that are not satisfactorily manageable) Data collection procedures can be refined and data recollected Other, more accurate data sources can be pursued
		Sampling bias	(see above)	(see above)	(see above)
		Label bias	(see above)	(see above)	(see above)
(e) Data features (internal)	Data	Inequality bias (*without* unfairness)	A dataset on health conditions in admitted patients exhibits higher rates of prostate cancer among male admitted patients than female ones	Asymmetry is between features of male population and female population within the dataset	Acceptable bias. No action needed
		Inequality bias (*with* unfairness)	A dataset on criminal recidivism in the U.S. (committing a second crime after a first, within a specified period of time) exhibits higher recidivism rates for African Americans than Caucasians	Asymmetry is between features of Caucasian population and African American population within the dataset	(i) Determine whether the data has been acquired in a way that constitutes **sampling bias**. If it *has*, then acquire a **more representative sample**; or supplement the existing sample with additional instances (real or synthetic) that correct the inequality. (ii) Determine whether the data has been acquired in a way that constitutes an **error bias**. If it *has*, then attempt to correct for the error. (iii) If the data hasn't been acquired in a way that introduces sampling or other error biases, conclude that the inequality in the dataset is an accurate representation of an **inequality in the environment**, and ask whether *this* inequality is fair or unfair. Be careful not to use any model trained on this data to guide decision-making or action such that unfairness in the environment is perpetuated by model-supported decisions or actions
(f) How data are processed by model	AI	Inductive bias	A neural network classifier trained on a particular dataset exhibits different output behavior depending on selected activation function, learning rate, update function (e.g. backpropagation), number of hidden layers, and other hyperparameters	Between one possible way of extrapolating from data and other possible ways of extrapolating from data.	Possibly acceptable bias. Researchers should reflect on the effects of any inductive biases (e.g. the effects of hyperparameter selection on classifier behavior) and, if time and resources permit, experiment with and explore the results from different sets of inductive assumptions
		Bias-variance trade-offs	Lasso regression is used to shrink coefficients and reduce influence of outliers on predictions	Between how datapoints with different features are treated by the model (e.g. outliers play less role in determining the shape of the regression line than they otherwise would)	Possibly acceptable bias. If time and resources permit, researchers should experiment with and explore the effects, on overall accuracy as well as (perhaps) accuracy within particular regions or for particular subtasks, of raising or lowering the level of bias of the type under consideration
(g) Model positionality (NL positionality) (Santy et al., 2023; Liu, 2024)	AI	“Worldview bias”	An LLM trained primarily on English-language sources may inadvertently exhibit individualist and pro-Western biases	Analogous to inductive bias for model selection. Asymmetry is between the model's performance and the performance of other models (or, the average performance of all possible models)	Likely unavoidable, yet consequential. Model positionality or “worldview bias” should be explored and documented, and the specific positionality or worldview bias of a model should be born in mind when deploying the model and interpreting its results
(h) Model features (internal)	AI	Inequality bias (*without* unfairness)	An LLM's language embeddings show stronger correlation between “male” and “prostate” than between “female” and “prostate”	Asymmetry is between two sets of (otherwise similar) model features (in this example, the tokens “male” and “female”)	Acceptable bias. No action needed
		Inequality bias (*with* unfairness)	An LLM's language embeddings show stronger correlation between “male” and “doctor” than between “female” and “doctor”	Asymmetry is between two sets of (otherwise similar) model features (in this example, the tokens “male” and “female”).	Unacceptable bias. Seek to identify source of bias (data? in-processing?) and select appropriate mitigation responses, which may include:(a) Retraining the model on a debiased dataset, (b) Reinforcement learning with human feedback (RLHF) to counteract the bias
		Process biases	Because of the procedures by which it was trained, an LLM is highly biased toward those answers that match the most frequently given answers in its training data, despite these being less relevant and accurate than other answers in the training data that are less frequently given	Analogous to cognitive biases in humans. The primary asymmetry is between an imagined or conceivable ideal process of operation, and the actual operation of the AI system	Sometimes acceptable, sometimes unacceptable. Whether process biases in a model's operation are acceptable usually depends on whether the *outputs* of the model are nonetheless accurate and reliable. However, identified process biases in a model's operation can be clues to conditions under which the model would fail, and models should be tested for their robustness under conditions that come to light in this way. Known process biases in a model's operation should be born in mind in all deployments of the model, including in deployments to guide decision-making or action. Process biases of a model that are known to affect performance should be explicitly stated by model producers (e.g. on model cards)
(i) How accurately the model represents	AI	Error biases	(a) An LLM's performance on questions about syntax of natural language (e.g. “How many ‘R's are in strawberry?”) exhibits lower accuracy than its performance on other questions due to its architecture	(a1) Asymmetry between the model's answers to some questions, and the reality that those answers are presented as describing (a2) Asymmetry between the model's performance on some types of questions (mostly accurate) and other types of questions (often inaccurate). [This type of bias reduces the *reliability* of the model, in addition to the costs of its erroneousness for the particular questions it answers incorrectly]	Undesirable but often inevitable for at least some conceivable use-cases of a model. Should be explicitly noted (e.g. on model cards) and (if possible) systematically tested and documented, with the range of expected failure precisely specified For each deployment context, consequences of failure should be studied and assessed for the severity of their risk Systems should not be used for tasks on which they show high risk of failure, nor used by themselves or primarily on any mission-critical tasks for which they show any significant risk of failure
			(b) A voice-recognition-powered transcription program sometimes erroneously translates spoken words. The erroneously transcribed words are often legal terminology, due to the transcription program's original use-cases and training for court transcription assistance	(b1) Asymmetry between the model's transcriptions and the actual spoken words these transcriptions are supposed to represent (b2) Asymmetry between the sub-vocabularies into which the system erroneously translates words (legal vs. non-legal)	
(j) Model's recommended decisions or actions (model outputs)	AI	Inequality biases(*without* unfairness)	A model designed to predict risk of prostate cancer exhibits a higher false positive rate for male patients than for female patients	Asymmetry is between model accuracy for male patients and model accuracy for female patients	Acceptable bias. No action needed
		Inequality biases(*with* unfairness)	A model designed to predict risk of recidivism in the U.S. (committing a second crime after a first, within a specified period of time) exhibits a higher false positive rates for African Americans than for Caucasians	Asymmetry is between model accuracy for Caucasians and model accuracy for African-Americans	Unacceptable bias. Reduce or eliminate use of the model until the unfair inequality in model outputs are corrected. Correction procedures may include: (a) If the unfair inequality is an inequality in model performance (e.g. accuracy), pursue technical solutions to increase accuracy for the less accurately scored subpopulation. (b) If the unfair inequality is an inequality in distribution of social goods, pursue post-processing correction procedures to make the distribution more equitable (unless such post-processing correction procedures would introduce more serious ethical problems)
(k) Decisions and/or beliefs based on the model and/or its output	AI or human or human-AI hybrid	Automation biases	Nursing staff at a hospital that has invested in AI-powered diagnostic equipment begins to favor equipments' diagnoses over that of human physicians, even in cases where the error rates of diagnostic equipment are significant and human oversight is recommended.	(a) Process bias. The nurses trust the AI answers relative to human answers more than they should, given the evidence. (b) Inequality bias. The nurses give more trust per unit of supporting evidence to the AI answers than they give to the human answers. (c) The inequality and process biases can easily lead, in this case, to error biases	Decision-makers should be taught and regularly reminded about limits of AI tools, seeking to bring their level of trust and investment into calibration with the trustworthiness of models
		Dismissal biases	Nursing staff at a hospital with AI-powered diagnostic equipment becomes tired of the upkeep involved in using it and begins to ignore its recommendations to avoid having to do this maintenance.	(a) Process bias. The nurses trust the human answers relative to AI answers more than they should, given the evidence. (b) Inequality bias. The nurses give more trust per unit of supporting evidence to the human answers than they give to the AI answers. (c) The inequality and process biases can easily lead, in this case, to error biases	Decision-makers should be taught and regularly reminded about relative benefits of AI tools, seeking to bring their level of trust and investment into calibration with the trustworthiness of these tools
		Availability bias	(see above)	(see above)	(see above)

In addition to these four major contributions, we draw on the new biases-as-asymmetries perspective to make two additional, incidental contributions: (a) new interpretations of commonly known cases of AI bias (Section 10) and (b) new interpretations of the increasingly relevant topic of LLM biases (Section 11).

Table 1 provides an overview of the paper's contents, organized by research contribution.

## 2 Are biases always bad?

One might think that whatever biases are, they must be some type of *error*. One commentator, for instance, defines “[c]ognitive bias” as “errors or flaws in judgment or decision-making, often to the point of denying reality,” and “a root cause of medical errors and sentinel events within the healthcare environment” (Stephens, 2019). Another notes that “to researchers trained in the psychometric tradition, the … term bias is practically synonymous with error, tending to connote general wrongness.” (1105) (Taniguchi et al., 2018).

It's also common to assume that all AI biases are *unfair*. A recent web source, for instance, writes that “Artificial intelligence (AI) bias occurs when a machine learning algorithm makes an error that leads to an unfair result” (WebFX, 2024). A recent systematic review defines “bias” as “systematic error in decision-making processes that results in unfair outcomes” (Ferraro, 2024).

Given these assumed links between “bias,” “error,” and “unfairness,” one might conclude that AI biases are always bad and should, as much as possible, be minimized or eliminated: “[b]oth popular and academic articles invariably present algorithmic bias as something bad that should be avoided” (Danks and London, 2017).

However, as many have recently argued, not all bias is a bad thing, and in fact is often necessary or desirable (Hagendorff and Fabi, 2023; Geman et al., 1992; Mitchell, 1980; Montanez et al., 2019; Taniguchi et al., 2018; Yarkoni and Westfall, 2017). A 2022 NIST special report on AI bias notes that “bias is not always a negative phenomenon” (Schwartz et al., 2022). A recent team of authors write that “bias can be a positive and desirable aspect of a well-engineered model when used to improve other model characteristics” (Landers and Behrend, 2023). Another argue that biases are essential to building AI systems that serve their intended purposes (Hagendorff and Fabi, 2023). How can these violations of the usual association between “bias” and “wrongness” be understood?

In our view, the key insight that explains why biases aren't always bad is that the term “bias” is more or less synonymous with “violation of a symmetry standard”: wherever anything, including an AI system, exhibits an asymmetry, that asymmetry can be described as a *bias* of a certain kind (Kelly, 2022). Since our world is not composed solely or even primarily of perfectly symmetrical objects and relationships, systems that were completely unbiased would be condemned to inaccurately represent important features of our world, making them less preferable to more biased alternatives that describe the world more accurately. Further, preferences themselves—including ethical valuations—are asymmetries. By failing to encode “asymmetrical” preferences for some things or outcomes over others, unbiased systems are less likely to be effective at guiding agents' actions and less likely to align with users' values, ambitions, and projects.

However, while a system's exhibiting *some* asymmetries rather than *no* asymmetries is almost always essential to its capacity for success, the precise asymmetries exhibited must be of a sort that improves rather than diminishes the system's accuracy and desirability over alternatives. Otherwise, the system is biased in a *bad* way—that is, its biases carry it off-course from accurately representing the world or effectively guiding our actions within it. These three insights—(1) biases are by definition just asymmetries, thus not inherently good or bad; (2) in almost all cases, *some* asymmetries in the system are essential to its functioning effectively; and (3) *which* asymmetries a system manifests make all the difference to whether that system performs as desired—are developed further in their application to AI systems below.

## 3 Defining “bias”

Recent discussions point the way to a definition of bias that applies to all or nearly all cases of bias, whether “good” or “bad.” This definition is focused on the notion of “asymmetry” (Kelly, 2022).

Hagendorff and Fabi propose that “a common denominator for all types of biases is that they can be paraphrased as some kind of distortion, as a tendency toward a particular value, as a specific presetting, or simply as deviation from a standard or a reduction of variety” (Hagendorff and Fabi, 2023). However, this definition is a compilation of heterogenous features that need not coincide. For instance, a process could involve distortion without tending to a particular value (as in high-noise or “high variance” samples); could tend to a particular value without that value being preset; or could deviate from a standard without reducing variety.

Danks and London give a clearer common formula: “‘bias' simply refers to deviation from a standard” (Danks and London, 2017). This definition's inclusivity is both a strength and a weakness: it can be used to describe cases of all types given in Hagendorff and Fabi's definition, but also includes things that don't fit any common or disciplinary-specific meaning of the word “bias.” For instance, any crime would count as a “bias” by this definition since it deviates from the standard of “legal behavior.” An athlete who fails to meet a performance standard in their tryout for a team could be described as giving a “biased” performance (even if, say, only 2% of those trying out make the team).

A more adequate definition is suggested by Kelly, who begins by claiming (like Danks and London) that “bias involves a systematic departure from a genuine norm or standard of correctness.” (4) (Kelly, 2022). Attributions of bias according to this meaning are always pejorative: that is, they always imply that bias is *bad*. However, Kelly then recognizes that our language also includes non-pejorative ascriptions of bias—for instance, the expression “a biased coin” (Kelly, 2022). These count as cases of bias, on his view, if they involve the breaking of some contextually relevant symmetry standard.

After recognizing the role of symmetry in non-pejorative bias ascriptions, Kelly comes to recognize the role of symmetry in the pejorative cases as well, suggesting that the extent to which violation of a norm is classifiable as *bias* correlates to the extent to which the violated norm in question is a symmetry standard: “paradigmatic instances of bias typically involve departures from standards that amount to symmetry violations, while being unbiased involves respecting or preserving certain symmetries and invariances.” (153) (Kelly, 2022).

For our purposes, we adopt Kelly's symmetry-based definition of bias, understanding this as equally applicable to pejorative and non-pejorative uses of the term. Thus, we define bias as “violation of a contextually relevant symmetry standard.”

**Figure 1:**
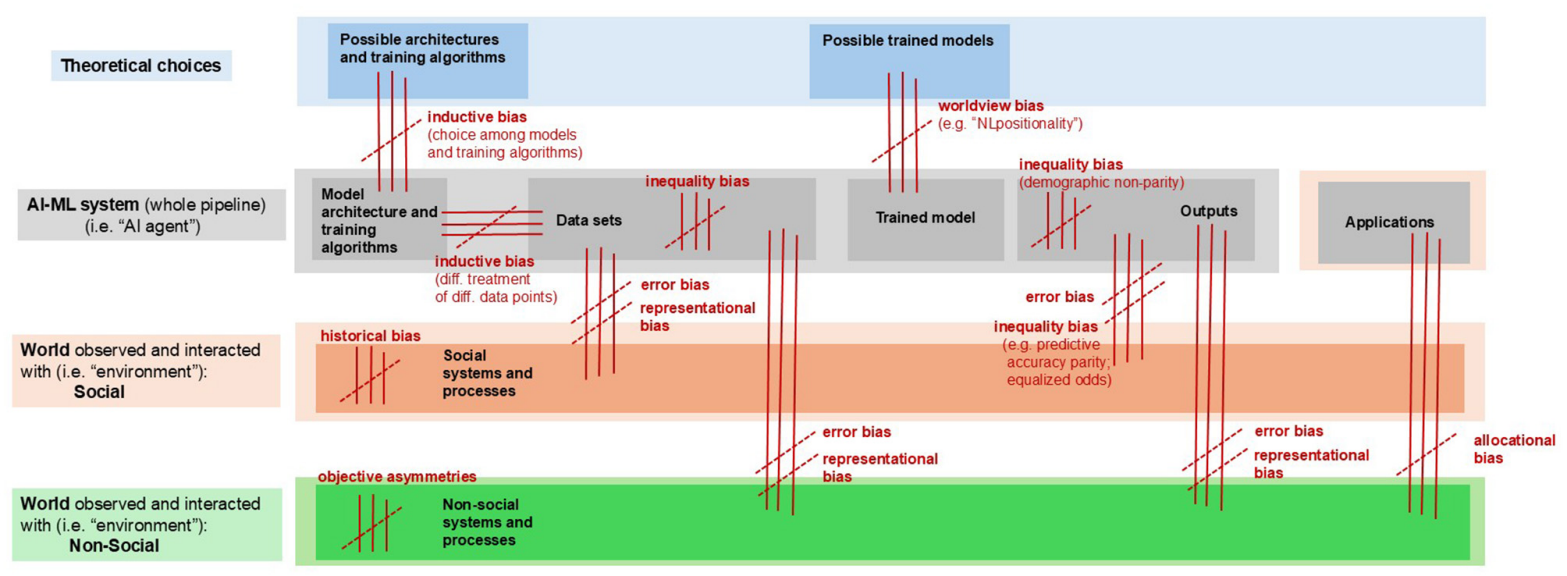
Common types of AI bias represented as asymmetries. Triple parallel lines indicate symmetries that are broken in cases of bias (axes of asymmetry may be perpendicular or parallel to these lines). The labels “error bias, “inequality bias,” etc., are defined further throughout the paper (e.g. Sections 5, 7, and Table 2). For “NLpositionality,” (see Santy et al., 2023).

This definition allows us to recognize several important, and too infrequently distinguished, biases (e.g. violations of symmetry standards) in play in artificial intelligence and machine learning contexts. These include (a) asymmetries in how data is collected; (b) asymmetries in how much data of various types is collected; (c) asymmetries in the content of that data; (d) asymmetries in how data points are handled by an interpreter (for instance, by an algorithm or theory); (e) asymmetries in how various parts of the world are represented by the resultant model (i.e. differential accuracy across different parts of the model's representational space); and (f) asymmetries in outputted decisions or classifications (judged by demographic parity or an accuracy-sensitive metric such as false positive rate or false negative rate across groups) (Carey and Wu, 2023; Dwork et al., 2012; Barocas et al., 2023; see Figure 1 for a visual map of asymmetries throughout the AI-ML lifecycle).

Two features of this list are especially noteworthy. First, not all of these asymmetries are *always* or even *usually* undesirable: asymmetries in how conclusions are drawn from data, for instance, is unavoidable in conditions of uncertainty (Mitchell, 1980; Montanez et al., 2019); and asymmetries in dataset *contents* is desirable where data are supposed to represent an asymmetrical reality. Second, a system can be biased in one of these ways without necessarily being biased in others. These two observations suggest that attributions of bias to a system should generally be accompanied by a specification of (1) what kind of symmetry standard is being violated in this case, and (2) whether the system's violation of that symmetry standard in that context is desirable or undesirable, and why.

Understanding biases as asymmetries newly illuminates common definitions in AI literature while connecting statistical, cognitive, and ethical perspectives. The asymmetry concept unifies these by highlighting a feature common to all forms of bias: breaking of some possible or expected symmetry.

## 4 Why are biases bad (When they are)?

If biases are just asymmetries, then some of the biases in AI systems are necessary or desirable. How then do we distinguish biases that are problematic and should be avoided or eliminated, from those that are innocuous or beneficial? (For ease of discussion, we will hereafter refer to the former category as “unacceptable biases,” and the latter as “acceptable biases”).

We propose that there are three main reasons that a bias can be unacceptable:

(1) The bias leads a system component to be *less accurate as a* representation of something it is supposed to represent;
(2) The bias leads to *unfair allocation of resources* between individuals or groups (these can include attentional and representational “resources”: thus, they include both of what (Suresh and Guttag, 2021) call “allocational” and “representational” biases);
(3) The bias involves a *deviation from processing ideals* that are themselves valid and applicable (for instance: canons of deductive logic or probability theory).

We will sometimes refer to these as “error biases,” “unfair inequality biases,” and “process biases,” respectively (explained further in Section 5 below).

Note that none of the just-listed three things is sufficient by itself to *define* bias, on our account. What *defines* bias is just asymmetry. But we propose that asymmetries are only unacceptable (when they are) for one of these three reasons. These three types of failure—failure of accuracy, failure of fairness, and failure of a process ideal (e.g. “rationality”)—define and delineate *reasons for finding a bias unacceptable* when we do. Thus, faced with evidence of bias in an AI system, and wanting to determine whether the bias is one that calls for correction or mitigation, we should ask three questions:

1. Is the bias *inaccurate* in any way that is unacceptable?
2. Is the bias *unfair* in any way that is unacceptable?
3. Does the bias involve a *deviation from process ideals* in any way that is unacceptable?

The traditional association of bias with “a regular pattern of error,” and the use of the term “bias” within the field of statistics, are especially concerned with the first of these three problems. Sometimes a bias involves outputs or internal components that do not represent reality accurately; they deviate from reality in some systematic way. Optical illusions, for instance, constitute biases of these kinds, as do biased samples in statistics.

Phrases such as “racial bias” and “gender bias” are almost always focused on the second of the problems, “unfair treatment.” These phrases signal that individuals from some groups (e.g. “African Americans,” “women”) are treated differently (asymmetrically) by comparison with individuals from other groups (e.g. “Caucasians,” “men”). Concerns about “algorithmic fairness” and “implicit bias” are likewise usually concerns about asymmetries of this type.

Finally, the literature on “cognitive biases” primarily focuses on problems of the third type: biases that lead the system to operationally deviate from a procedural ideal. However, it is worth noting that deviations from one processing ideal may sometimes involve greater adherence to a *different* processing ideal. In these cases, the system may indeed be “unacceptable” by the first processing standard, but “acceptable” by the second processing standard. These observations echo the somewhat paradoxical result of decades of research into cognitive “heuristics and biases” (Tversky and Kahneman, 1974): namely, that such biases bring a variety of benefits and costs to cognitive processes, which depend heavily on the circumstances in which they're employed (Gigerenzer and Brighton, 2009; Hafenbraedl et al., 2016; Taniguchi et al., 2018).

Bias in “inductive bias” and “bias-variance tradeoffs,” discussed further below, often do not generate problems of the three types noted; thus, these likewise concerns ways that data processing deviates from some processing ideals. But, like cognitive biases, these deviations sometimes bring the system closer to *other* processing ideals that are ultimately more contributory to accuracy than those that are violated (and the “bias” in such systems is often itself a source of *error*—just not, in cases where more “bias” is recommendable over more “variance”—as *much* a source of error as the variance that is thereby reduced).

## 5 Error biases, inequality biases, and process biases

As a shorthand expression for biases that are potentially unacceptable for one of the three reasons given above, we will sometimes refer to these as “error biases,” “unfair inequality biases,” and “process biases.” These terms refer to cases where an asymmetry involves systematic deviations from representational accuracy, differential treatment that counts as unfairness, or performance that counts as violation of a processing ideal, respectively.

Note that biases of these three types—inaccuracy biases, unfair inequality biases, and process biases—cannot simply be classified as unacceptable in *every* case that they appear, but rather must be considered in context. For instance: in some cases, a certain degree of inaccuracy is inevitable and the badness of this is offset by other benefits of the system. The question of whether a particular unfair inequality, inaccuracy, or process bias is acceptable or unacceptable is often an ethically complex one where specific features of the case should be considered in making a decision. Our purpose in delineating these three types of bias as the main sources of unacceptability in AI biases is *not* to offer a one-step operational criterion that allows all cases of unacceptable bias to be identified and classified by reference to that criterion alone. It is rather to give guidance on what kind of evidence to look for, and what kinds of arguments to make, when deciding about what AI biases to accept or reject. We argue that when an AI bias is legitimately found unacceptable, it is almost always for at least one of these three reasons (we thank a reviewer for pointing out the possible confusion).

Error biases and process biases are *prima facie* unacceptable insofar as they involve violation of ideals that normally apply in all cases (accuracy and processing ideals, respectively). But there are cases where increases of error bias or process bias can reduce overall error (“bias-variance tradeoffs” and “cognitive biases and heuristics,” respectively, discussed further below).

The category of “inequality bias” technically applies to every case of bias (including error and process biases) insofar as inequality is a synonym of “asymmetry.” However, some cases of inequality count as “unfair,” thus triggering the second main reason why biases can be unacceptable when they are. Hence it is useful to distinguish “inequality biases without unfairness” from “inequality biases with unfairness” (i.e. “unfair inequality biases”). And even unfair inequality biases may sometimes be acceptable (for instance: when the benefits of employing the system apply even to the “worst off” in an unfair distribution, and no less unfair system is feasible: see Rawls, 1974 for elaboration).

## 6 Can biases be unacceptable for more than one reason?

Biases sometimes fit into two or more of the main problem types simultaneously. For instance, suppose a non-profit organization providing food assistance has explicitly adopted, as a processing standard, that all incoming applications for emergency assistance should be ranked first by severity of need, then by proximity to the non-profit's storage facilities, in determining order of response. A review of the organization's practices reveals that applications from some locations were immediately placed at the bottom of the list, regardless of severity of need. This asymmetry of treatment based on geographical location is a violation of both the stated processing ideal and a possible principle of equal response for all applications.

To take another example: suppose a model of consumer preferences is trained on a very imbalanced dataset, such that consumers in some demographic groups (e.g. women) are represented in a way that deviates significantly from the actual distribution of behavior in the larger population of that demographic group. The deviation itself is a case of sampling error, and thus a case of representational error. But the difference in accuracy between men and women that results constitutes a case of unfairness as well.

If cases of bias can fall into more than one of these three problem categories, does the distinction between them still hold value? We believe that it does. Distinguishing and identifying the precise reasons that a case of bias is problematic is essential to determining what should be done in response. To take an analogous case: there are different kinds of “rights” that humans can have (for instance: legal rights and human rights). Many cases that involve a violation of one of these rights also involve violations of others. Nonetheless, it is analytically valuable—and in some cases essential—to distinguish different kinds of rights that have been violated in a particular case, as a step toward identifying the actions that should be taken to protect and restore those rights.

Indeed, once we acknowledge that biases are not inherently unacceptable but only unacceptable under certain conditions, it becomes extremely important to develop a clearly articulated and applicable terminology for distinguishing unacceptable biases from others. A more fitting analogy, then, is with a category such as “transfer of property,” which sometimes occurs by legitimate processes (e.g. gifts, purchase, taxation) and sometimes by illegitimate ones (e.g. larceny, embezzlement, extortion). There may be cases where property was transferred in ways that include multiple of these illegitimate categories at once (e.g. larceny and extortion); it is still important to have clear standards for what counts as a case of each type of illegitimate form of transfer, wherever it occurs.

## 7 Four sometimes good and sometimes bad biases

In this section we look more closely at a few commonly discussed types of AI bias. We interpret these in terms of the type of asymmetry they exhibit, and show that their “acceptability” or “unacceptability” varies based on context and can be better understood from this perspective.

(a) *Unequal treatment biases*

It is important to recognize that while all cases of unequal treatment are cases of bias, not all of these cases are problematic or undesirable. For instance: “Biased” allocation of medical resources based on differences in risk factors among patients involves unequal treatment, but this “biased” allocation is exactly what we want the system to do, since we want it to allocate resources to those who need them and not waste resources on those who don't. In these cases, minimizing “error bias” and achieving our goals for the system can *only* be accomplished through unequal treatment.

When an unequal treatment bias is deemed unacceptable, the reason is usually that the bias seems to be *unfair*. Discussions of “racial bias” and “gender bias” in AI are usually about fairness in this sense, as are most discussions of algorithmic fairness and fair AI-ML (e.g. Buolamwini and Gebru, 2018; Singh et al., 2024). Well-known statistical tests for fairness such as demographic parity, false positive rate parity, and predictive accuracy parity (Carey and Wu, 2023; Dwork et al., 2012; Barocas et al., 2023), are often used to estimate the extent to which unequal treatment biases of various kinds characterize a system's decision-making. These metrics differ from one another in what *kind* of equality or inequality of treatment they measure (for instance: equal distribution of a trait between groups [“*independence”*]; equal rates of false positives or false negatives between groups [“*separation”*]; or equal predictive power of assigned scores to individuals in both groups [“*sufficiency”*]) (Carey and Wu, 2023; Dwork et al., 2012; Barocas et al., 2023).

A remarkable feature of these fairness metrics is that in many cases they cannot be simultaneously satisfied (Kleinberg et al., 2016; Chouldechova, 2017). Thus it is sometimes necessary for a decision-making system to be “unfair” by at least one standard of fairness that legitimately applies to some cases. Since no-one should be asked or expected to do the impossible, the “fairness impossibility” result highlights the need to be careful and deliberate about choosing the fairness standards by which one evaluates a system (Honenberger, 2025). AI developers should carefully consider which fairness criteria are most appropriate for their specific use case and be transparent about the tradeoffs involved. For instance: proportional parity (“independence”) is likely preferable in circumstances where resources should be allocated completely independently of subject features, such as distribution of stimulus payments to individual citizens in a stressed national economy (Kearns and Roth, 2019). Reduction of false positive or false negative rate imbalance (“separation”) may be preferable when false positives or false negatives are particularly damaging or ethically unacceptable, such as deciding on anti-suicide interventions or eligibility for pre-trial release or a loan. Maximation of predictive accuracy parity (“sufficiency”) may be the best fit when overall accuracy is the primary target and errors are not especially harmful, such as advertising or recommender systems (Honenberger, 2025).

(b) *Inductive biases*

Mitchell (1980) provides an early example of an argument that biases are necessary for informative inductive inferences [though his reasoning parallels Goodman (Goodman, 1955) and Hume (Hume, 1739). This idea is subsequently discussed as “inductive bias.” Mitchell (1980) writes that “we use the term bias to refer to any basis for choosing one generalization over another, other than strict consistency with the observed training instances.” He then argues that consistency with the observed training instances will never be enough to select among alternative generalizations within what he calls the “version space” of possible generalizations. The generalization that is identical to the training instances, on the other hand, provides no means to predict any data points beyond what's already given in those instances. Thus, biases are necessary as means to break the tie between otherwise equivalently possible generalizations within the version space; without such a bias, induction itself would be impossible. Mitchell points to some specific kinds of biases that can be useful for selecting the generalization, such as “[f]actual knowledge of the domain,” “[i]ntended use of the learned generalizations,” “[k]nowledge about the source of training data,” “[b]ias toward simplicity and generality,” and “[a]nalogy with previously learned generalizations” (Mitchell, 1980). Mitchell's discussion focuses on systems that “learn” to generalize on the basis of training data, but a parallel argument can be made for any inductive system.

Subsequent arguments for the necessity of bias in inductive inferences follow a similar trajectory as Mitchell's, often with citations to his 1980 paper (Wilson and Martinez, 1997; Montanez et al., 2019).

Notice the very limited sense of “bias” according to which such systems are shown to be biased in these arguments: namely, they are biased in their selection of an inductive procedure or conclusion *from among all possible procedures or conclusions consistent with the data alone* (the “possible architectures and training algorithms” step, and the link between model and data set, in Figure 1). If, on the other hand, we examine the choice of inductive procedure from a standpoint that *includes* the supplementary information (for instance, domain knowledge), then the choice may be very *un*biased by relevant *processing* standards, insofar as it tracks relevant differences between the available hypotheses.

Likewise, if we consider these “biased” inductions by the standard of accuracy to their representational targets—that is, their “error bias” (in Figure 1, this is the error biases associated with “outputs”)—then we see that “theory-choice-biased” inductions are *more* capable than “unbiased” alternatives of being representationally *un*biased inductions—a fact implied by the authors' description of theory-choice-biased inductions as potentially *more accurate* (that is, more accurate as descriptors or predictors of their representational targets).

Furthermore, while these arguments show that biased inductions *can be* more accurate than completely unbiased ones (insofar as completely unbiased inductions fail to classify or predict future instances that differ from the training instances in any way), they don't show that more biased inductions are *in general* more accurate than less biased ones; nor even that *all* biased inductions are an improvement over completely unbiased ones. Consider that an induction that misclassifies or falsely predicts future datapoints is hardly better, and possibly worse, than one that refuses to classify or predict at all. At least one possible generalization in what Mitchell calls the “version space” will have this “gets *every* new datapoint wrong” feature; thus, at least one available “biased” induction is arguably as bad or worse than a completely unbiased induction.

(c) *Cognitive biases*

The literature on “cognitive biases” describes processes of reasoning or inference that have suboptimal results in many cases, or deviate from canons of rationality—for instance: principles of first-order logic or Bayesian probability theory.

However, since Tversky and Kahneman, the terms “cognitive bias” and “heuristics” have often been used interchangeably (Tversky and Kahneman, 1974); and while “cognitive bias” carries some implication of error or at least deviation from optimal rationality, “heuristics,” in its use by Simon and others, implies a shortcut that is nonetheless effective at problem solving (Simon and Newell, 1958). Research has shown that certain cognitive biases can enhance performance in specific contexts (Taniguchi et al., 2018). For example, the “availability heuristic” bias, which prioritizes easily recalled information, can be beneficial for rapid decision-making in emergency situations (Tversky and Kahneman, 1973).

One might suppose heuristics can contribute efficiency but only at the expense of accuracy. However, some have argued that cognitive biases and heuristics are actually optimal for *accuracy* in some circumstances, particularly those in which the application context is very different from the training context (Gigerenzer, 1991; Gigerenzer and Brighton, 2009; Hafenbraedl et al., 2016; Hjeij and Vilks, 2023). The relative benefits and costs of a more biased system, in these circumstances, are similar to those between higher-bias-lower-variance systems and higher-variance-lower-bias systems in bias-variance tradeoffs (discussed further below).

It is important to note that, in cases where higher bias is found to be beneficial, not just any bias will achieve the benefit. The bias must be in a direction and of a type and quantity that improves overall accuracy (even if the system is still inaccurate in many of its particular decisions or predictions, and even if it is this way *because of* the bias). This means that *some* cognitive biases, in *some* circumstances, reduce “error bias,” and are therefore “good” or “preferable” in those circumstances. But not *every* cognitive bias is good or preferable in *every* circumstance. The particular cognitive bias *and* the particular context are of essential importance in determining whether a particular cognitive bias is an overall beneficial feature of a system.

(d) *Bias in bias-variance tradeoffs*

The “bias-variance” tradeoff has long been known to machine learning practitioners (Geman et al., 1992). Here “bias” means “systematic deviation of predicted values from actual values” and is contrasted with “variance,” which signifies a random deviation of predicted values from actual values. The discovered “trade-off” is that sometimes increasing the bias can decrease the variance, and sometimes this can result in lower overall inaccuracy than if the bias were lower but variance were higher. In other words: sometimes higher-bias models exhibit lower variance than lower-bias alternatives, and this reduction in variance can lead to higher overall accuracy for the model (Geman et al., 1992; Gigerenzer and Brighton, 2009; Yarkoni and Westfall, 2017).

Geman et al. (1992) introduced this idea of a bias-variance trade-off. Beginning with a contrast between *non-parametric* (“model free”) and *parametric* (“model dependent”) approaches to statistical inference tasks, they argued that the estimation error of a statistical inference procedure could be decomposed into two main components: bias and variance. Roughly put, variance is random and non-directional error, whereas bias is systematic error (typically directional, though perhaps in different directions for different parts of a dataset or learning task). Model-free architectures tend to have low bias but high variance, while model-dependent architectures exhibit lower variance but higher bias. Since all statistical inference procedures are somewhere on a spectrum between model-free and model-dependent, all such procedures can be located in a space of tradeoffs between bias and variance.

In some circumstances, the risk and extent of estimation errors arising from variance is greater than that arising from bias. Introducing bias into such systems can reduce variance and, in many cases, reduce overall error as a result. Simple examples of such variance-reducing but bias-increasing procedures include eliminating outliers; using drop-out (Srivastava et al., 2014); reducing the parameter size of the resulting model (e.g. requiring the model function to be no greater than an n-term polynomial); and stopping the training procedure before overfitting occurs. In circumstances where more error derives from variance than bias, such procedures can improve predictive accuracy overall.

Features of learning environments wherein variance is a major challenge to accuracy include: low quantity of training data; noisy data; and prediction or application contexts that are relatively unlikely to be like the training context (Gigerenzer and Brighton, 2009; Hafenbraedl et al., 2016). In these environments, a system that overfits will be drastically out of step with its representational target for any new applications (interpolations, extrapolations, or otherwise). A more biased system, however, can keep this variance problem in check by holding predictive values within a range less likely to be wildly inaccurate.

This line of thinking supports the conclusion that more biased inductions are *sometimes* more accurate than less biased ones. However, several qualifications of this conclusion should be noted.

First, *the precise bias that is selected makes an enormous difference*. In a case where variance is a problem and introduction of biases can help, some biases that could be selected will reduce variance more than they generate new sources of error. But, in almost all such cases, some others of the biases that could be selected will reduce variance but *also* generate greater sources of error than they eliminated (now due to bias). Without any reason to choose one bias over another, we risk making things worse rather than better with any bias we choose (for the same reasons noted earlier in connection with “cognitive biases” and “inductive biases”). Further, when we have a reason to choose one bias rather than another, this *choice* of bias is itself evidentially motivated, and hence shows elision of at least some kinds of potential process bias.

Second, *the benefit of the bias in bias-variance tradeoffs is merely relative and instrumental*: if a model that were equivalently lower variance but *also* lower bias became available, that lower-bias-and-lower-variance model would generally be preferable to the high-bias model. This shows that the epistemic goodness of increases in such biases is merely an instrumental goodness (tied to reduction in variance).

## 8 What should be done about AI biases (and why)?

Previous discussions of AI bias often classify biases by the stage of the AI lifecycle at which they appear: for instance, biases in datasets, in model selection, in output, and in applications (Danks and London, 2017). Combining this common approach with the asymmetry-based classification of AI biases developed in previous sections provides a novel systematic framework for understanding and evaluating AI biases. This framework enables us to more clearly distinguish biases that are acceptable from those that are unacceptable, as well as to think about trade-offs between biases of different types (Table 2).

Table 2 gives a classification of AI biases by (a) the type of asymmetry that defines them and (b) the stage of the development cycle at which they appear. This allows us to note (c) which types of biases are acceptable or unacceptable, and make recommendations for “mitigation” accordingly.

We can separate the biases described in this table into three categories: *Necessary (in some regard)*; *Usually good*; and *Usually bad*. When biases are necessary or good, they are usually acceptable; when biases are bad, they are usually unacceptable (we say “usually” because of complications such as biases that are bad in one way but good in another. In these cases, deciding on their acceptability or unacceptability requires attention to specifics of the case).

(A) *Necessary biases*

For the reasons discussed above, *inductive biases* in the stages of model selection, in-processing, outputs, and applications are almost always a necessary condition of carrying out an induction (a prediction or classification) at all. However, the *precise* biases selected or exhibited make an enormous difference to whether the results will be good or bad. In this sense, some specific inductive biases are better than others, and some are quite bad. Which specific biases are better or worse depends heavily on features of the context.

Likewise, *unequal treatment* in datasets, in-processing, outputs, and applications is often necessary as well, for two basic reasons. First, datasets often contain evidence of bias in the systems they provide information about. Indeed, a completely symmetrical dataset is likely to be relatively uninformative. Second, when it comes to in-processing, outputs, and applications, the fact that “fairness” can be measured by competing and incompatible metrics (“fairness impossibility”) means that at least one of the incompatible metrics will be violated in almost all cases.

What should be done about unavoidable biases of these two types?

For inductive biases, we recommend (a) accepting that some inductive bias is unavoidable, but (b) trying to select the inductive bias that maximizes accuracy, fairness, and other goals of the induction.

For biased datasets, we recommend evaluation of the biases in light of the functions the datasets will be used for. For instance: a model designed to accurately measure an asymmetrical reality generally *ought* to be biased in the same way and to the same extent that the represented reality is “biased.” However, when models are used to inform decision-making at the “application” step, such biases can provide grounds for differential treatment that is ultimately unfair. Bias in datasets must therefore be handled delicately, in context of the full use cycle of data selection, training, and deployment.

In general, unequal treatment biases at the in-processing, outputs, or applications stages that are at risk of being unfair should be vetted via the fairness metrics that appropriately apply to them. This requires carefully selecting the metrics that should apply in each case, and, in cases where these are mutually incompatible, carefully deciding which to prioritize and to what extent (Honenberger, 2025).

(B) *Usually good biases*

In general, biases of an AI are “good” (when they are) for one of three reasons:

(1) the bias in the AI system *represents* a bias in the system modeled (i.e. the system's bias effectively tracks asymmetries in the world that the AI system is being used with the intention of tracking). Examples include inductive biases, cognitive biases, and some inequality biases.
  In these cases, the bias in that part of the model should generally be preserved; however, the added value of the bias for *representation* purposes should be quarantined from any unfair effects on *actions* (recommended or selected).
(2) the bias in the AI system *reduces noise* in the modeling of the system modeled (i.e. reduces *variance*), thereby improving accuracy, as recognized in the bias-variance trade-off. In these cases, the selection of a higher bias model may be justified; but the researcher should also (i) continue to explore the possibility of simultaneously lower-bias and lower-variance models; and (ii) seek an explanation for the success of the higher-bias model in this case that can help guide the search for simultaneously lower-bias and lower-variance models.
(3) the bias in the AI system tracks *preference* asymmetries—for instance, by ranking items of some kinds higher than others, or by differentially responding to individuals in a manner than corrects for or repairs previous unfairness biases. These can be described as process biases and (often) as inequality biases, but ones that track adopted preferences, and may be overall desirable.

In all of these cases, we encourage preservation or amplification of the useful bias, so long as attention to other possible biases resulting from the current model and their total costs (as well as the costs of other possible undesirable effects of the model) are borne in mind. The useful bias should not create more serious problems than it solves.

(C) *Usually bad biases*

In general, biases of an AI are “bad” (when they are) for one of three basic reasons:

(1) the bias in the AI system *distorts or deviates from an accurate representation* of the systems it is used with the intention to represent (“error biases”);
(2) the bias in the AI system *distorts or deviates from a fair representation* of the systems it is used to make decisions about (“unfair inequality biases”);
(3) the bias in the AI system comes at a *cost to other desirable features* of the AI system (e.g. its rationality; efficiency; the trust assigned to it by the public; etc.)

In cases of “bad” biases by any three of these standards, the developer has a number of options, including:

(i) *eliminate* the bias (for instance, via supplemented *datasets* or *in-processing* procedures)
(ii) *mitigate* the bias (for instance, via post-processing at the end of in-processing or the outputs stage; or an adjusted application procedure)
(iii) *accept* the bias as a “best case scenario” or “necessary evil” (for instance: if an analysis in terms of “bias-variance” tradeoff or fairness impossibility reveals it to be so).

## 9 Detecting and mitigating problematic asymmetries in AI systems

The discussion of bias mitigation is necessarily changed once we acknowledge that the badness of AI biases cannot be assumed. In many cases, the appropriate response to bias is not to do anything at all (e.g. inequality biases without unfairness). In others, it is to be aware of the bias and report it but not necessarily to eliminate it (e.g. inductive biases). In yet others, it is to undertake mitigation of the traditional kinds, in an effort to bring the system to a less unfair, erroneous, or otherwise problematic state (Feldman and Peake, 2021; Siddique et al., 2024; Cary et al., 2023).

Understanding AI biases as asymmetries brings clarity and flexibility to the practice of AI bias mitigation within this new theoretical context. Empirically grounded approaches to identifying and responding to biases throughout the AI development cycle include the following:

(1) Bias detection

Effective detection of asymmetries often requires multifaceted approaches that vary according to the stage of development and the type of bias being examined:

*Dataset analysis and visualization* (Chawla et al., 2002; Denis et al., 2024; DiCiccio et al., 2023; Wang et al., 2022): statistical techniques for detecting representational and demographic disparities in training data may include diversity indices, demographic parity metrics, and correlation analyses between protected attributes and target variables. Visualization tools can reveal hidden patterns of demographic imbalance or feature correlations that might otherwise remain obscure.

*Fairness metrics* (Singh et al., 2024; DiCiccio et al., 2023; Wang et al., 2022): formal statistical metrics provide quantitative measures of inequality biases. These include demographic parity (comparing prediction rates across groups), equalized odds (comparing false positive and true positive rates across groups), and predictive parity (comparing precision across groups). The selection of appropriate metrics depends heavily on the specific context and purpose of the AI system as these metrics cannot be satisfied simultaneously in most cases.

*Adversarial testing*: probing potential biases through targeted queries or scenarios designed to elicit problematic responses can reveal asymmetries not detectable through standard testing procedures. For instance, replacing demographically associated names, locations, or other attributes in input data can reveal whether the system responds differently based on these attributes (Idemudia, 2023).

*Explainability tools*: techniques like SHAP (Shapley Additive exPlanations) and LIME (Local Interpretable Model-agnostic Explanations) can indicate features contributing most significantly to model decisions to help detect when protected attributes or their proxies are unduly influencing outputs (Salih et al., 2024). Recent work has explored using constructed knowledge graphs to infer the presence, and even the multi-step pathways, of biases throughout ML pipelines, starting from datasets but including biases introduced during in-processing (Russo et al., 2024).

*Bias detection in complex systems*: for large complex AI systems such as LLMs, specialized approaches include bias benchmarks (standardized test datasets designed to elicit biased responses), representation testing (examining the distributional characteristics of embeddings), and counterfactual fairness assessments (measuring how outputs change when protected attributes are altered) (Coppolillo et al., 2025).

(2) Bias mitigation

Effective mitigation of problematic asymmetries typically employes techniques targeted to specific stages of the AI development cycle:

(i) *Preprocessing techniques* (Chawla et al., 2002; Romano et al., 2020):
  (a) *Balanced dataset creation*: addressing inequality or error biases through sampling techniques (oversampling underrepresented groups or undersampling overrepresented ones), synthetic data generation, or data augmentation.
  (b) *Feature transformation*: removing or transforming problematic features and their proxies, applying techniques like fair representation learning that preserves predictive power while minimizing correlations with protected attributes.
  (c) *Counterfactual data augmentation*: creating balanced datasets by generating counterfactual examples that alter protected attributes while preserving other relevant features.
(ii) *In-processing techniques*:
  (a) *Fairness constraints*: incorporating fairness objectives directly into the learning algorithm's optimization process, either through regularization terms or constrained optimization approaches (Raza et al., 2024; Denis et al., 2024).
  (b) *Adversarial debiasing*: training the model to maximize predictive performance while simultaneously minimizing the ability to predict protected attributes from its representations (Reimers et al., 2021).
  (c) *Self-supervised pre-training*: for deep learning systems, using self-supervised learning on diverse datasets before task-specific fine tuning can improve representation quality for underrepresented groups (Marks et al., 2025).
(iii) *Post processing techniques*:
  (a) *Output calibration*: adjusting model predictions to achieve fairness metrics such as equalizing error rates across groups or ensuring demographic parity (DiCiccio et al., 2023; Romano et al., 2020).
  (b) *Ensemble methods*: combining multiple models trained with different objectives or on different subsets of data to balance competing fairness and accuracy constraints (Feffer et al., 2022).
  (c) *Selective neuron modification*: in neural network-based systems, identifying and modifying activations of specific neurons that encode problematic biases (Liu et al., 2022).

It is important to note that these techniques involve tradeoffs. Mitigation strategies that address one type of bias might exacerbate others or might reduce predictive accuracy. The selection and implementation of appropriate detection and mitigation techniques should be guided by careful consideration of the specific context, stakeholder needs, and ethical implications of the system being developed.

## 10 Examples of asymmetries in deployed AI systems

The framework developed in this paper helps to illuminate real-world cases of AI bias by identifying the specific asymmetries at work, the stages at which they arise, and whether they represent problematic or beneficial deviations from symmetry standards. Below are several examples:

*Recruitment algorithms*: amazon's experimental recruiting tool exhibited gender bias by penalizing resumes containing terms associated with women (e.g., “women's chess club”) and favoring language patterns more common in men's resumes (Dastin, 2018). This represents an error bias at the output stage (the model inaccurately associated gender with qualification) and an inequality bias in applications (differential treatment of candidates based on gender). The bias originated from historical data reflecting past hiring patterns that favored men, which illustrates how dataset asymmetries can propagate through the AI pipeline.

*Criminal justice risk assessment*: the COMPAS recidivism prediction algorithm demonstrated higher false positive rates for Black defendants compared to white defendants (Angwin et al., 2016). While the system achieved similar overall accuracy across racial groups (satisfying the “sufficiency” fairness criterion), it violated the “separation” criterion by imposing different error costs on different demographic groups. Unfair inequality biases at the output stage can manifest even when overall predictive accuracy appears balanced as in this example.

*Computer vision systems*: facial recognition technologies have exhibited substantially higher error rates for women with darker skin tones compared to men with lighter skin tones (Buolamwini and Gebru, 2018). This represents an inequality bias in outputs, with error rates varying dramatically across demographic groups. The bias originated from imbalanced training data overrepresenting certain demographics, combined with algorithmic choices that failed to account for this imbalance. This phenomenon demonstrates how biases can compound across multiple stages of the AI pipeline.

*Recommendation systems*: content recommendation algorithms on social media platforms have been shown to exhibit filter bubble effects where users receive increasingly narrow content aligned with their existing preferences (Parisi, 2011; Ludwig et al., 2024). This represents a process bias at the application stage that can create feedback loops that can potentially reinforce existing biases. While the initial asymmetry in content selection might be intended to enhance user experience (a “good bias”), the cumulative effect can lead to problematic information asymmetries and social polarization.

These examples illustrate several insights from the proposed framework:

(a) Biases often propagate and transform across stages of the AI development cycle
(b) The same system can exhibit multiple types of asymmetries simultaneously
(c) Context is crucial in determining whether a particular asymmetry is problematic
(d) Addressing one type of bias may require tradeoffs with other types of bias or other desirable or undesirable properties.

## 11 Biases in large language models

Large Language Models (LLMs) represent a particularly challenging domain for bias analysis due to their scale, complexity, and generative capabilities (Bender et al., 2021; Gallegos et al., 2024; Lee et al., 2024). Our framework of understanding biases as asymmetries provides insights into the nature and management of biases in these systems.

LLMs exhibit distinct patterns of asymmetry at each stage of development. In the data collection stage, the vast web-scraped corpora used to train LLMs contain numerous asymmetries that reflect historical and contemporary societal biases. These include overrepresentation of certain languages, cultures, and perspectives; imbalanced representation of demographic groups; and asymmetric associations between demographic identifiers and attributes. Unlike traditional supervised learning datasets, these asymmetries are more difficult to detect and quantify due to the scale and heterogeneity of the data.

At the same time, the basic functionality and usability of LLMs, like other machine learning models, relies on asymmetries learned from its data: the word embeddings and attention heads that support transformer models are themselves a record of asymmetries in word co-occurrence across various linguistic contexts (Vaswani et al., 2017; Bender et al., 2021).

LLM architectures encode specific inductive biases through their attention mechanisms, parameter sharing, and optimization procedures. These architectural choices enable the generalization capabilities of LLMs, but also shape how they extrapolate from training data to novel inputs. Self-supervised training objectives (e.g., next token prediction) create additional process biases that prioritize statistical regularities over common human communicative ideals such as factual accuracy and ethical considerations. Common methods of fine-tuning pre-trained transformers, such as reinforcement learning through human feedback (RLHF) or constitutional fine-tuning, may introduce additional biases associated with user preferences or the programmers' values.

LLMs also exhibit numerous asymmetries in their outputs, including generation of text that perpetuates stereotypical associations between demographic groups and attributes (e.g., occupations, traits, behaviors). Even after years of large-scale bias mitigation efforts, these associations sometimes resurface, manifesting themselves in unexpected ways (Hofmann et al., 2024). LLMs have also been found to produce higher quality outputs for dominant languages and dialects by comparison with less represented ones (Bender et al., 2021); present information with different levels of certainty, nuance, and accuracy depending on the topic or entities involved; and exhibit preferences or aversions that align more with some demographics than others (Santurkar et al., 2023; Santy et al., 2023).

When LLMs are deployed in applications, additional asymmetries emerge in how they serve different user groups and purposes. These include disparities in service quality across languages, cultures, and domains of expertise; differential capabilities to recognize and accommodate the needs of users from various backgrounds; and varying access based on geography, technological literacy, and income.

Our asymmetry-based framework newly illuminates several aspects of bias in LLMs. First, some asymmetries in LLMs are necessary for their functioning. For example, inductive bias toward linguistic coherence is essential for generating readable text. However, asymmetries that amplify social inequities or produce factually incorrect representation call for intervention.

Second, traditional bias detection methods often fail with LLMs due to their generative nature, entropy in user inputs, and vast parameter space. Evaluating asymmetries across diverse inputs and contexts requires specialized approaches like prompt-based probing, counterfactual testing, and holistic evaluations across multiple dimensions of fairness and accuracy. The universal yet flexible framework of biases-as-asymmetries may provide unique resources for conceptual and practical navigation of the complex bias-space of LLMs across their many possible contexts of use. Strategies for addressing problematic asymmetries in LLMs include diverse data curation, balanced fine-tuning datasets, adversarial training techniques, and post-processing methods that align model outputs with ethical and equitable standards. These approaches can involve tradeoffs between reducing certain asymmetries and maintaining model performance, or introducing new asymmetries.

Third, LLMs introduce novel fairness concerns that are not adequately captured by traditional metrics. Their ability to generate content rather than classifying it requires expanding the conceptualization of fairness to include considerations such as stereotypical associations, representation quality, and differential impacts across cultural contexts.

The field of LLM development illustrates the importance of distinguishing between beneficial, necessary, and problematic asymmetries. Future research could focus on developing more nuanced frameworks for evaluating the complex interplay of asymmetries in these systems and designing targeted interventions that preserve beneficial asymmetries while mitigating harmful ones.

## 12 Conclusions

We began with three questions: (1) How should we think about and measure biases in AI systems, consistent with the understanding that biases aren't necessarily bad? (2) What kinds of bias in an AI system should we accept, and why? and (3) what kinds of bias in an AI system should we not accept, and why?

In answer to (1), we've argued that “biases” should be conceived as asymmetries (Sections 2-3). The definition of bias as asymmetry, drawn from Kelly (Kelly, 2022), applies both to acceptable and unacceptable forms of bias.

In answer to (2) and (3), we distinguished three potential sources of unacceptability in AI biases (Sections 4–7): *erroneous representation* (where outputs deviate from real-world referents), *unfair treatment* (where decision processes disadvantage some individuals or groups more than others, with no legitimate reason for the different treatment), and *violation of process ideals* (for instance, where rationality is compromised). We further considered major types of asymmetry throughout the AI development-to-application lifecycle, and noted which types of bias, at which stages in this process, are especially likely to be acceptable or unacceptable (Sections 8, 9, 11, and Table 2).

The approach to AI biases proposed here has several implications for AI development and evaluation. By categorizing biases based on both their lifecycle stage and asymmetry type, developers can better identify and address biases at each stage of AI development, as well as distinguish acceptable from unacceptable biases. This approach not only aids in identifying and mitigating unacceptable biases, but also in optimizing acceptable biases to enhance AI performance and fairness. For instance, distinguishing representative from anti-representative biases in datasets can guide the development of more representative datasets. Distinguishing ineliminable inductive biases and unfairness biases from optional and variable ones reduces the risk of accepting harmful biases that might otherwise have been avoided. Finally, acknowledging tradeoffs between biases of different types clarifies that choices about which symmetries to preserve or disrupt are almost never selections between “biased” and “unbiased” processes, but rather between processes that are biased in different ways, some of which are acceptable and some of which are unacceptable, for a variety of reasons and dependent on the context.

Future research directions for a symmetry-based approach to AI biases include (i) development of quantitative measures for each type of bias identified in the typology; (ii) investigation of relations of amplification or trade-off between different types of biases; and (iii) exploration of how this typology can inform regulatory frameworks for AI governance. We leave these tasks for future efforts.

In the field of AI bias mitigation and elsewhere, the assumption that “all bias is bad” seems to be giving way to a pragmatic recognition that systems *must* be biased in some ways if they are to fulfill the various functions that we require of them. This recognition raises a new danger, however, that warnings about problematic, harmful, or otherwise unacceptable biases will go unheeded since “bias, after all, is inevitable and often good.” The analysis given here, if understood, cuts such “universal” defenses of AI biases off at the pass by showing that the distinction between acceptable and unacceptable biases *can* be made for clear and convincing reasons. It thus sets the stage for a new and more effective bias mitigation regime in the coming generation.

## References

[B1] Angwin J. Larson J. Mattu S. Kirchner L. (2016). Machine Bias. ProPublica, May 2016.

[B2] Barocas S. Narayanan A. Hardt M. (2023). Fairness in Machine Learning: Limitations and Opportunities. Cambridge, MA: MIT Press.

[B3] Bender E. Gebru T. McMillan-Major A. Schmitchell S. (2021). On the Dangers of stochastic parrots: can language models be too big? Proc. FAccT 2021, 610–623. 10.1145/3442188.3445922

[B4] Buolamwini J. Gebru T. (2018). “Gender shades: intersectional accuracy disparities in commercial gender classification,” in Proceedings of Machine Learning Research, 81, 1–15. Available online at: https://proceedings.mlr.press/v81/buolamwini18a/buolamwini18a.pdf

[B5] Carey A. Wu X. (2023). The statistical fairness field guide: perspectives from social and formal sciences. AI Ethics 3, 1–23. 10.1007/s43681-022-00183-3 35574571 PMC9099231

[B6] Cary M. P. Zink A. Wei S. Olson A. Yan M. Senior R.. (2023). Mitigating Racial and ethnic bias and advancing health equity in clinical algorithms: a scoping review. Health Equity 42:10. 10.1377/hlthaff.2023.00553 37782868 PMC10668606

[B7] Chawla N. Bowyer K. Hall L. Kegelmeyer P. (2002). SMOTE: synthetic minority over-sampling technique. J. Artif. Int. Res. 16, 321–357. 10.1613/jair.953

[B8] Chouldechova A. (2017). Fair prediction with disparate impact. Big Data 5, 153–163. 10.1089/big.2016.0047 28632438

[B9] Coppolillo E. Manco G. Aiello L. M. (2025). Unmasking conversational bias in AI multiagent systems. arXiv [Preprint] arxiv:2501.14844. 10.48550/arxiv.2501.14844 PMC13484959 42611856

[B10] Danks D. London A. (2017). “Algorithmic bias in autonomous systems,” in Proceedings of the 26th International Joint Conference on Artificial Intelligence (IJCAI 2017) (New York, NY: ACM). 10.24963/ijcai.2017/654

[B11] Dastin J. (2018). Insight - Amazon Scraps Secret AI Recruiting Tool That Showed Bias Against Women. Reuters. Available online at: https://www.reuters.com/article/world/insight-amazon-scraps-secret-ai-recruiting-tool-that-showed-bias-against-women-idUSKCN1MK0AG/ (Accessed August 29, 2025).

[B12] Denis C. Elie R. Hebiri M. Hu F. (2024). Fairness guarantees in multi-class classification with demographic parity. J. Mach. Learn. Res. 25, 1–46. Available online at: https://www.jmlr.org/papers/volume25/23-0322/23-0322.pdf (Accessed September 9, 2025).

[B13] DiCiccio C. Hsu B. Yu Y. Nandy P. Basu K. (2023). “Detection and mitigation of algorithmic bias via predictive parity,” in Proceedings of the 2023 ACM Conference on Fairness, Accountability, and Transparency (FAccT '23) (New York, NY: Association for Computing Machinery), 1801–1816. 10.1145/3593013.3594117

[B14] Dwork C. Hardt M. Pitassi T. Reingold O. Zemel R. (2012). “Fairness through awareness,” in Proceedings of the 3rd Innovations in Theoretical Computer Science Conference (New York, NY: ACM), 21–30. 10.1145/2090236.2090255

[B15] Feffer M. Hirzel M. Hoffman S. Kate K. Ram P. Shinnar A. (2022). Navigating ensemble configurations for algorithmic fairness. arXiv [Preprint] arXiv:2210.05594. 10.48550/arXiv.2210.05594

[B16] Feldman T. Peake A. (2021). End-to-end bias mitigation: removing gender bias in deep learning. arXiv [Preprint] arXiv:2104.02532. 10.48550/arXiv.2104.02532

[B17] Ferraro E. (2024). Fairness and bias in artificial intelligence: a brief survey of sources, impacts, and mitigation strategies. Sci 6:3. 10.3390/sci6010003

[B18] Gallegos I. O. Rossi R. A. Barrow J. Tanjim M. M. Kim S. Dernoncourt F.. (2024). Bias and fairness in large language models: a survey. Comput. Linguist. 50, 1097–1179. 10.1162/coli_a_00524

[B19] Geman S. Bienenstock E. Doursat R. (1992). Neural networks and the bias/variance dilemma. Neural Comput. 4, 1–58. 10.1162/neco.1992.4.1.118249860

[B20] Gigerenzer G. (1991). How to make cognitive illusions disappear: beyond ‘heuristics and biases'. Eur. Rev. Soc. Psychol. 2, 83–115. 10.1080/14792779143000033

[B21] Gigerenzer G. Brighton H. (2009). Homo heuristicus: why biased minds make better inferences. Topics Cogn. Sci. 1, 107–143. 10.1111/j.1756-8765.2008.01006.x 25164802

[B22] Goodman N. (1955). “The new riddle of induction,” in Fact, Fiction, and Forecast (Cambridge, MA: Harvard University Press).

[B23] Hafenbraedl S. Waeger D. Marewski J. Gigerenzer G. (2016). Applied decision making with fast-and-frugal heuristics. J. Appl. Res. Memory Cogn. 5, 215–231. 10.1016/j.jarmac.2016.04.011

[B24] Hagendorff T. Fabi S. (2023). Why we need biased AI: how including cognitive biases can enhance AI systems. J. Exp. Theor. Artif. Intell. 36, 1885–1898. 10.1080/0952813X.2023.217851740294189

[B25] Hjeij M. Vilks A. (2023). A brief history of heuristics: how did research on heuristics evolve? Humanit. Soc. Sci. Commun. 10:64. 10.1057/s41599-023-01542-z

[B26] Hofmann V. Kalluri P. R. Jurafsky D. King S. (2024). AI generates covertly racist decisions about people based on their dialect. Nature 633, 147–154. 10.1038/s41586-024-07856-5 39198640 PMC11374696

[B27] Honenberger P. (2025). “Fairness impossibility in AI-ML: an integrative ethics approach,” in Philosophy of AI: the State of the Art, eds. V. C. Müller, A. R. Dewey, L. Dung, and G. Löhr (Springer). Forthcoming.

[B28] Hume D. (1739) [1975]. A Treatise of Human Nature. Oxford: Clarendon Press. 10.1093/oseo/instance.00046221.

[B29] Idemudia I. (2023). Red teaming: a framework for developing ethical AI systems. Am. J. Eng. Res. 12, 07–14. Available online at: https://www.ajer.org/papers/Vol-12-issue-10/12100714.pdf (Accessed September 9, 2025).

[B30] Kearns M. Roth A. (2019). The Ethical Algorithm. Oxford: Oxford University Press.

[B31] Kelly T. (2022). Bias: A Philosophical Study. Oxford: Oxford University Press. 10.1093/oso/9780192842954.001.0001

[B32] Kleinberg E. Mullainathan S. Raghavan M. (2016). Inherent trade-offs in the fair determination of risk scores. a*rXiv* [Preprint]. arXiv:1609.05807. 10.48550/arXiv.1609.05807

[B33] Landers R. N. Behrend T. S. (2023). Auditing the AI auditors: a framework for evaluating fairness and bias in high stakes AI predictive models. Am. Psychol. 78, 36–49. 10.1037/amp0000972 35157476

[B34] Lee J. Hicke Y. Yu R. Brooks C. Kizilcec R. F. (2024). The life cycle of large language models in education: a framework for understanding sources of bias. Br. J. Educ. Technol. 55:13505. 10.1111/bjet.13505

[B35] Liu H. (2024). Worldview of the AICG systems: stability, tendency, and polarization. AI Soc. 40, 2493–2506. 10.1007/s00146-024-01966-4

[B36] Liu S. Reviriego P. Lombardi F. (2022). Selective neuron re-computation (SNRC) for error-tolerant neural networks. IEEE Trans. Comput. 71, 684–695. 10.1109/TC.2021.3056992

[B37] Ludwig K. Mueller P. Nikolajevic N. Grote A. (2024). Putting ‘filter bubble' effects to the test: evidence on the polarizing impact of ideology-based news recommendations from two experiments in Germany and the U.S. Inf. Commun. Soc. 10.1080/1369118X.2024.2435998

[B38] Marks M. Knott M. Kondapaneni N. Cole E. Defraeye T. Perez-Cruz F.. (2025). A closer look at benchmarking self-supervised pre-training with image classification. Int J Comput Vis. 133, 5013–5025. 10.1007/s11263-025-02402-w 40727247 PMC12289721

[B39] Mitchell T. (1980). “The need for biases in learning generalizations,” in Rutgers CS Tech Report CBM-TR-117. Available online at: https://www.cs.cmu.edu/~tom/pubs/NeedForBias_1980.pdf (Accessed August 29, 2025).

[B40] Montanez G. Hayase J. Lauw J. Macias D. Trikha A. Vendemiatti J.. (2019). The futility of bias-free learning and search. arXiv [Preprint] arXiv:1907.06010. 10.48550/arXiv.1907.06010

[B41] Parisi E. (2011). The Filter Bubble: What the Internet is Hiding From You. Penguin Press.

[B42] Rawls J. (1974). A Theory of Justice. Cambridge, MA: Harvard University Press.

[B43] Raza S. Raval A. Chatrath V. (2024). MBIAS: mitigating bias in large language models while retaining context. arXiv [Preprint]. arXiv:2405.11290v2. 10.48550/arXiv.2405.11290v2

[B44] Reimers C. Bodesheim P. Runge J. Denzler J. (2021). “Conditional adversarial debiasing: towards learning unbiased classifiers from biased data,” in Pattern Recognition. DAGM GCPR. (2021). Lecture Notes in Computer Science, Vol 13024, eds. C. Bauckhage, J. Gall, and A. Schwing (Cham: Springer), 48–62. 10.1007/978-3-030-92659-5_4

[B45] Romano Y. Bates S. Candes E. (2020). “Achieving equalized odds by resampling sensitive attributes,” in Proceedings of Neural Information Processing Systems Available online at: https://proceedings.neurips.cc/paper/2020/file/03593ce517feac573fdaafa6dcedef61-Paper.pdf (Accessed September 9, 2025).

[B46] Russo M. Chudasama Y. Purohit D. Sawischa S. Vidal M.-E. (2024). Employing hybrid AI systems to trace and document bias in ML pipelines. IEEE Access 12, 96821–96847. 10.1109/ACCESS.2024.3427388

[B47] Salih A. M. Raisi-Estabragh, Z. Galazzo I. B. Radeva P. Petersen S. E. Lekadir K. Menegaz G. (2024). A perspective on explainable artificial intelligence methods: SHAP and LIME. Adv. Intell. Syst. 7:2400304. 10.1002/aisy.202400304

[B48] Santurkar S. Durmus E. Ladhak F. Lee C. Liang P. Hashimoto T.. (2023). Whose opinions do language models reflect? *arXiv* [Preprint]. arXiv: 2303.17548. 10.48550/arXiv:2303.17548

[B49] Santy S. Liang J. T. Le Bras R. Reinecke K. Sap M. (2023). NLPositionality: characterizing design biases of datasets and models. a*rXiv* [Preprint]. arXiv: 2306.01943. 10.48550/arXiv:2306.01943

[B50] Schwartz R. Vassilev A. Greene K. Perine L. Burt A. Hall P.. (2022). Towards a Standard for Identifying and Managing Bias in Artificial Intelligence. Gaithersburg, MD: NIST Special Publication (NIST SP). 10.6028/NIST.SP.1270

[B51] Siddique S. Haque M. A. George R. Gupta K. D. Gupta D. Faruk M. J. H.. (2024). Survey on machine learning biases and mitigation techniques. Digital 4, 1–68. 10.3390/digital4010001

[B52] Simon H. Newell A. (1958). Heuristic problem solving: the next advance in operations research. Oper. Res. 6, 1–10. 10.1287/opre.6.1.140577811

[B53] Singh N. Kapoor A. Soni N. (2024). A sociotechnical perspective for explicit unfairness mitigation techniques for algorithm fairness. Int. J. Inf. Manag. Data Insights 2:100259. 10.1016/j.jjimei.2024.100259

[B54] Srivastava N. Hinton G. Krizhevsky A. Sutskever I. Salakhutdinov R. (2014). Dropout: a simple way to prevent neural networks from overfitting. J. Mach. Learn. Res. 15, 1929–1958. Available online at: https://jmlr.org/papers/v15/srivastava14a.html (Accessed September 29, 2025).33259321

[B55] Stephens M. (2019). Cognitive debiasing: learning to ‘change your mind'. Nurse Leader 18, 344–351. 10.1016/j.mnl.2019.03.013

[B56] Suresh H. Guttag J. (2021). “A framework for understanding sources of harm throughout the machine learning lifecycle,” in EAAMO'21 (New York, NY: ACM). 10.1145/3465416.3483305

[B57] Taniguchi H. Sato H. Shirakawa T. (2018). A machine learning model with human cognitive biases capable of learning from small and biased datasets. Nat. Sci. Rep. 8:7397. 10.1038/s41598-018-25679-z 29743630 PMC5943317

[B58] Tversky A. Kahneman D. (1973). Availability: a heuristic for judging frequency and probability. Cogn. Psychol. 5, 207–232. 10.1016/0010-0285(73)90033-9

[B59] Tversky A. Kahneman D. (1974). Judgment under uncertainty: heuristics and biases. Science 185, 1124–1131. 10.1126/science.185.4157.1124 17835457

[B60] Vaswani A. Shazeer N. Parmar N. Uszkoreit J. Jones L. Gomez A. N.. (2017). Attention is all you need. ArXiv. Available online at: https://arxiv.org/html/1706.03762

[B61] Wang A. Liu A. Zhang R. Kleiman A. Kim L. Zhao D.. (2022). REVISE: a tool for measuring and mitigating bias in visual datasets. Int. J. Comput. Vis 130, 1790–1810. 10.1007/s11263-022-01625-5

[B62] WebFX (2024). What is AI Bias?: How to Detect and Prevent AI Bias. Available online at: https://www.webfx.com/digital-marketing/glossary/what-is-ai-bias/ (Accessed September 4, 2024).

[B63] Wilson D. R. Martinez T. (1997). “Bias and the probability of generalization,” in Proceedings of the 1997 International Conference on Intelligent Information Systems (Grand Bahama Island: IEEE), 108–114. 10.1109/IIS.1997.645199

[B64] Yarkoni T. Westfall J. (2017). Choosing prediction over explanation in psychology: lessons from machine learning. Perspect. Psychol. Sci. 12, 1100–1122. 10.1177/1745691617693393 28841086 PMC6603289

